# Overview of Native Chicken Breeds in Italy: Conservation Status and Rearing Systems in Use

**DOI:** 10.3390/ani11020490

**Published:** 2021-02-12

**Authors:** Annelisse Castillo, Marta Gariglio, Alessandro Franzoni, Dominga Soglia, Stefano Sartore, Arianna Buccioni, Federica Mannelli, Martino Cassandro, Filippo Cendron, Cesare Castellini, Alice Cartoni Mancinelli, Nicolaia Iaffaldano, Michele Di Iorio, Margherita Marzoni, Sonia Salvucci, Silvia Cerolini, Luisa Zaniboni, Achille Schiavone

**Affiliations:** 1 Dipartimento di Scienze Veterinarie, Università degli Studi di Torino, Largo Paolo Braccini 2, 10095 Grugliasco, Italy; annelisse.castillogarrido@unito.it (A.C.); marta.gariglio@unito.it (M.G.); alessandro.franzoni@unito.it (A.F.); dominga.soglia@unito.it (D.S.); stefano.sartore@unito.it (S.S.); 2 Dipartimento di Scienze e Tecnologie Agrarie, Alimentari, Ambientali e Forestali, Università di Firenze, Via delle Cascine 5, 50144 Firenze, Italy; arianna.buccioni@unifi.it (A.B.); federica.mannelli@unifi.it (F.M.); 3 Department of Agronomy, Food, Natural Resources, Animals and Environment (DAFNAE), University of Padova, Viale dell’Università 16, 35020 Legnaro, Italy; martino.cassandro@unipd.it (M.C.); filippo.cendron@phd.unipd.it (F.C.); 4 Dipartimento di Scienze Agrarie, Alimentari e Ambientali, Università di Perugia, Borgo XX Giugno 74, 06121 Perugia, Italy; cesare.castellini@unipg.it (C.C.); alice.cartonimancinelli@unipg.it (A.C.M.); 5 Dipartimento Agricoltura, Ambiente e Alimenti, Università degli Studi del Molise, Via Francesco De Sanctis, 86100 Campobasso, Italy; nicolaia@unimol.it (N.I.); michele.diiorio@unimol.it (M.D.I.); 6 Dipartimento di Scienze Veterinarie, Università di Pisa, Viale delle Piagge 2, 56124 Pisa, Italy; margherita.marzoni@unipi.it (M.M.); sonia.salvucci@unipi.it (S.S.); 7 Dipartimento di Medicina Veterinaria, Università degli Studi di Milano, Via dell’Università 6, 26900 Lodi, Italy; silvia.cerolini@unimi.it (S.C.); luisa.zaniboni@unimi.it (L.Z.)

**Keywords:** Italian poultry breeds, avian biodiversity, autochthonous poultry

## Abstract

**Simple Summary:**

The ongoing loss of domestic animal breeds around the world is occurring at an alarming rate. Thus, the registration and preservation of native breeds is of great importance. The aim of this study, which forms part of a conservation program, was to provide an overview of the conservation statuses of native Italian poultry breeds being reared by local breeders in Italy. The data collected by means of a census questionnaire demonstrate the low population sizes of these breeds in Italy and highlight the need for campaigns aimed at publicizing and promoting the benefits of native breeds with the goal of increasing population sizes. Identifying strategies to facilitate breeders’ access to pure breed birds is also essential, and would require collaborative efforts of university research centers, public entities, and breeders.

**Abstract:**

The most reared species of farm animal around the world is the chicken. However, the intensification of livestock systems has led to a gradual increase in the concentration of a limited number of breeds, resulting in substantial erosion to the genetic pool. The initial step of an ‘animal conservation program’ entails establishing the actual conservation statuses of the breeds concerned in a defined area; in this case, in Italy. To this end, a survey of breeds was performed by means of a census questionnaire divided into two parts. The first part collected information on breeds, breeders, housing facilities, and management aspects, the results of which are presented here. The second part of the questionnaire regarded chicken products and their markets, and these data will be reported in a second paper. The breed status of six chicken breeds was shown to be exceptionally worrying, with total numbers ranging from just 18 to 186 birds. Population sizes exceeding 1000 birds was identified for just four breeds, the maximum being 3400. Some improvements in status were noted in relation to breeds which had been the subject of conservation efforts in the past. The two most common breeds reported are the Bionda Piemontese, a double-purpose breed, and the Livorno egg-laying hen. Collo Nudo Italiano, Millefiori Piemontese, Pollo Trentino, and Tirolese chicken breeds and the Castano Precoce turkey breed were not listed by breeders at all. The most reported turkey breeds are the Bronzato Comune and the Ermellinato di Rovigo. The population sizes of native Italian poultry breeds were shown to be generally poor. Italian poultry farmers and the population at large are largely ignorant about indigenous poultry breeds. Thus, promoting the virtues of Italian breeds would help their conservation by encouraging breeders to rear these birds and consumers to buy their products. The identification of strategies to facilitate access to pure breed birds is essential, and will require the collaboration of university research centers, public entities, and breeders. The results presented in this paper constitute the initial part of a more complex conservation program.

## 1. Introduction

The demand for poultry products continues to grow and is reflected by steady increases in their output. One negative consequence of this trend, however, has been the preference for high yielding commercial hybrids, leading to drastic reductions in the farming of local breeds. Indeed, with the pressures of globalized economies on production yields, the farming of local breeds, which is characterized by more limited production outputs, has undergone significant decline. Furthermore, requirements for product uniformity and stringent food hygiene standards have limited the potential for small-scale poultry breeders to commercialize their products [1]. That said, trends change, and thankfully the productivity of a breed is not the sole factor influencing the choices of many modern-day farmers, breeders, and consumers. Indeed, the valorization of a breed should embrace values that go beyond economic aspects, and include elements such as cultural, socioeconomic, and environmental values [2].

The genetic characterizing of breeds and description of the overall picture regarding local realities constitutes an important part of the management of farm animal genetic resources. According to the Food and Agriculture Organization (FAO) [3], 53% of native breeds of farmed and domesticated animals are at risk of extinction in Europe and the Caucasus. In Italy, 53 local chicken breeds have been recognized [4], of which 67% are now extinct and 21% are at risk of extinction [3]. In fact, FAO has ranked the conservation status of 18 Italian chicken breeds as endangered or critically endangered [3].

As in other developed countries, safeguarding the biodiversity of native poultry breeds is becoming a matter of great concern. Over the last decades, conservation programs of local chicken breeds have been developed in cooperation with local and regional institutions in the regions of Lombardy [5], Veneto [6,7,8], and Emilia Romagna [9]. In recent years, a National Registry including 22 native chicken breeds was created and breed standards approved as part of a large cross-sectional Conservation Project being conducted by the Italian Ministry of Agricultural, Food and Forestry Policies (MIPAAFT), associated with Ministerial Decree No. 1936 of the 1 October 2014 [10]. Additionally, the numerous research papers available on this issue demonstrate the interest and work being directed towards the protection of these Italian breeds. Proteomic characterization and genetic studies addressing the issues of diversity, breed characterization, and molecular markers have been conducted in relation to the following breeds: Ancona [11,12,13], Bianca di Saluzzo and Bionda Piemontese [14,15,16,17,18], Ermellinata di Rovigo [7,19,20,21,22,23,24], Livorno [11,12,13,15,25], Mericanel della Brianza [15,26,27], Milanino [15], Millefiori di Lonigo [19], Modenese [11,12], Padovana and Pepoi [7,19,20,22,23,24], Polverara [7,19,20], Robusta Lionata [7,19,22,24], Robusta Maculata [19,20,22,24], Romagnola [11,12], Siciliana [15], Valdarnese Bianca [11,12,25], and turkey breeds [28,29].

Studies on breeding, productive performance, product quality, rearing management, welfare, and physiological traits are also available on the following breeds: Ancona [30,31,32,33,34], Bianca di Saluzzo [35], Bionda Piemontese [35,36,37], Ermellinata di Rovigo [6,38,39,40,41,42], Livorno [43,44,45], Mericanel della Brianza [46,47,48], Milanino [5,49,50,51,52], Modenese [9,11,53], Mugellese [54], Padovana [55,56,57,58,59], Polverara [55,57,60], Robusta Lionata [39,61], Robusta Maculata [6,36,37,38,39,40,41,42], Romagnola [9,53,62], Siciliana [44], and Valdarnese Bianca [36,37].

According to the Italian National Veterinary Service [63], the current number of registered free-range chicken farms in Italy housing less than 250 birds each is 1095, involving a total of 54,314 birds. Bigger farms, housing more than 250 birds, number 4610, for a total of over 135 million birds. The number of registered fancy breeder farms is 505, of which 442 house less than 250 birds. The whole overall turkey population comprises more than 11 million birds, distributed across 801 farms, most of which hold more than 250 birds, and only 31 farms constitute small farms. The number of birds belonging to native Italian breeds within these farms is unknown [63].

Despite the efforts made until now, there is still a long way to go to reduce the risk of significant loss to the genetic pool of Italian poultry breeds. In order to execute a project aimed at safeguarding farm animal biodiversity, an updated database on poultry breeds must first be created [64]. As part of a more complex program, which also includes characterizing the genomic variability of native Italian poultry breeds [65], the aim of this study was to collect information by means of a census questionnaire on the native breed population sizes, the rearing systems employed, and whether the rearing of native Italian breeds constitutes their keepers’ primary or secondary occupation.

## 2. Materials and Methods

A questionnaire was designed as a part of a large cross-sectional project called ‘Conservation of biodiversity in Italian poultry breeds’ [66], which focuses on safeguarding, conserving and improving the genetic resources of Italian poultry, i.e., the native breeds historically present in the country and included in the MIPAAF Registry of the Native Poultry Breeds [10].

**Figure 1:**
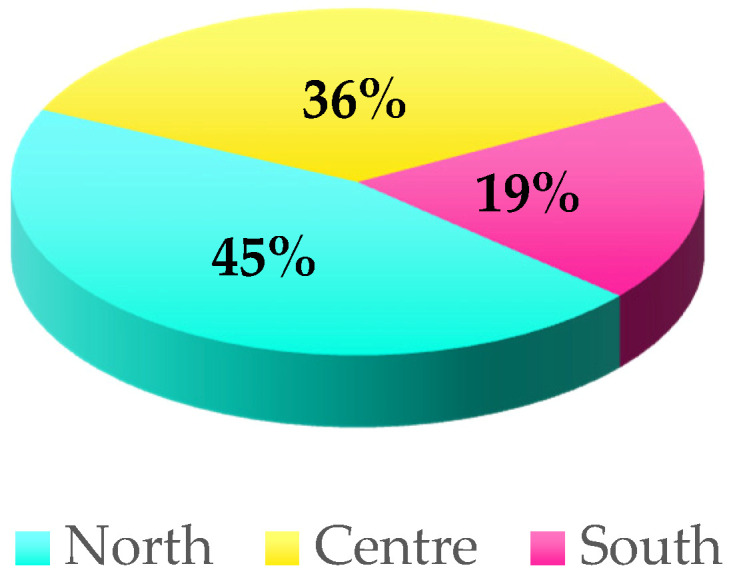
Italian breeders’ distribution by region.

The questionnaire, which focuses on native Italian chicken and turkey breeds, was devised to evaluate population sizes, housing conditions, management practices, and the product production according to breeder categories: farmers (F) and fancy breeders (FB), the former referring to farmers rearing birds on a commercial scale, and the latter referring to those keeping chickens as backyard poultry. The questionnaire consisted of closed and semiclosed questions and was divided into two parts. The first part included: the personal information pertaining to the breeders themselves; the chicken and turkey breeds reared; housing conditions and furnishings; nutrition, health; biosecurity. The second part was designed to gather information on chicken products produced from Italian local breeds and their market. The second part was developed to evaluate meat and table-egg production and their respective markets. A pilot test of the questionnaire was conducted on local farms in the Piedmont region, in the north-west of Italy [67] to improve the survey and make it as clear as possible; the data collected as part of the pilot test are not included in the present study. The questionnaire included breeders from North, Central and South Italian regions (Figure 1).

This study reports outcomes of the first part of the questionnaire, a subsequent paper will present the results of the second part.

A comprehensive list of Italian native breed poultry farmers and fancy breeders and their contact information was created by compiling lists from various sources, such as regional farmer associations and national and local fancy breeder associations. Breeders with more than 10 animals of each native breed were invited to fill in a questionnaire by means of face-to-face interviews conducted by researchers. Data were collected between June 2018 and June 2019, and researchers evaluated the existing flocks of each breed and sizes.

After each farm visit, data were entered into a purpose-made Microsoft Office Excel spreadsheet [68], using manual double entry and data entry checked for errors. JMP 9.0.1 software [69] was used for all statistical analyses. The chi-squared test, followed by the Fisher’s test, was used to determine significant differences in the distribution of variables between and within the two breeder categories: farmers and fancy breeders. *p*-values less than 0.05 were considered as statistically significant. Results are presented as the number and percentage of farmers and fancy breeders for each categorical variable. For certain variables, the sum of the responses obtained from the two breeder categories together did not necessarily equal the total number of breeders, this may have arisen due to nonresponses, or reflected the fact a response to some questions was only required depending on how a previous question had been answered.

## 3. Results

A total of 121 breeders participated in the study. Figure 1 reports their distribution by region. The North include Piemonte, Valle d’Aosta, Liguria, Lombardia, Trentino-Alto Adige, Veneto, Friuli-Venezia Giulia, and Emilia-Romagna. The Center include Toscana, Umbria, Marche, Lazio, and Sardegna. The South include Abruzzo, Molise, Campania, Puglia, Basilicata, Calabria, and Sicilia. Description statistics for the two breeder categories, regarding breeder gender, age, and whether their rearing activities constituted their main or secondary occupation, are reported in Table 1. The majority of breeders (62%) belonged to the F category (*p* < 0.01). Over three quarters were male (77% vs. 23%, *p* < 0.01), and the majority of breeders of both genders fell into the 30–50 and 50–70 age ranges (*p* < 0.01). This trend was also observed for females belonging to the F category (*p* < 0.01), whereas most males in the F category were aged 50–70 years (54%, *p* < 0.01). In relation to FB, no significant differences in age distribution were observed for either gender (*p* > 0.05). In both breeder categories, the rearing of native poultry breeds was mainly a secondary job (F 68% and FB 93%, *p* < 0.01). Moreover, on 76% of farms (F and FB), birds were exclusively managed by family members (Table S1), and in 95% of cases, a total of no more than four family members were involved in the related farming activities. In farms where external personnel were involved, in 60% of cases, the number of employees was less than 5.

The subsequent sections report the responses from the 121 surveyed Italian breeders on the following issues: breeds reared, poultry-house design and furnishings, bird nutrition, flock health, and biosecurity (procedures used to prevent or reduce disease hazards).

### 3.1. Bird Species and Population Sizes According to Breeder Category

Table 2 reports the data gathered on the native Italian bird species being reared. Data pertaining to the total sample (i.e., all breeders) are shown as well as divided according to breeder category. The results for the total sample show that more breeders’ rear chickens only than chickens plus other bird species (57% vs. 43%, *p* < 0.05). The same trend was also observed in FB (61% vs. 39.13%, *p* < 0.05), whereas no significant difference was detected for F.

Independently of breeder category, on the 52 farms rearing poultry species other than chickens, the percentage of farms also rearing turkeys was the greatest (58%), followed by those rearing ducks (44%), geese (42%) and Guinea fowl (42%, *p* < 0.01). Equal allocation was observed in F for these species (*p* < 0.01). The same was also true with respect to FB, except for geese which were reared to a lesser degree (39%, *p* < 0.01). Turkeys (78%) were highly preferred by FB (*p* < 0.01).

**Figure 2:**
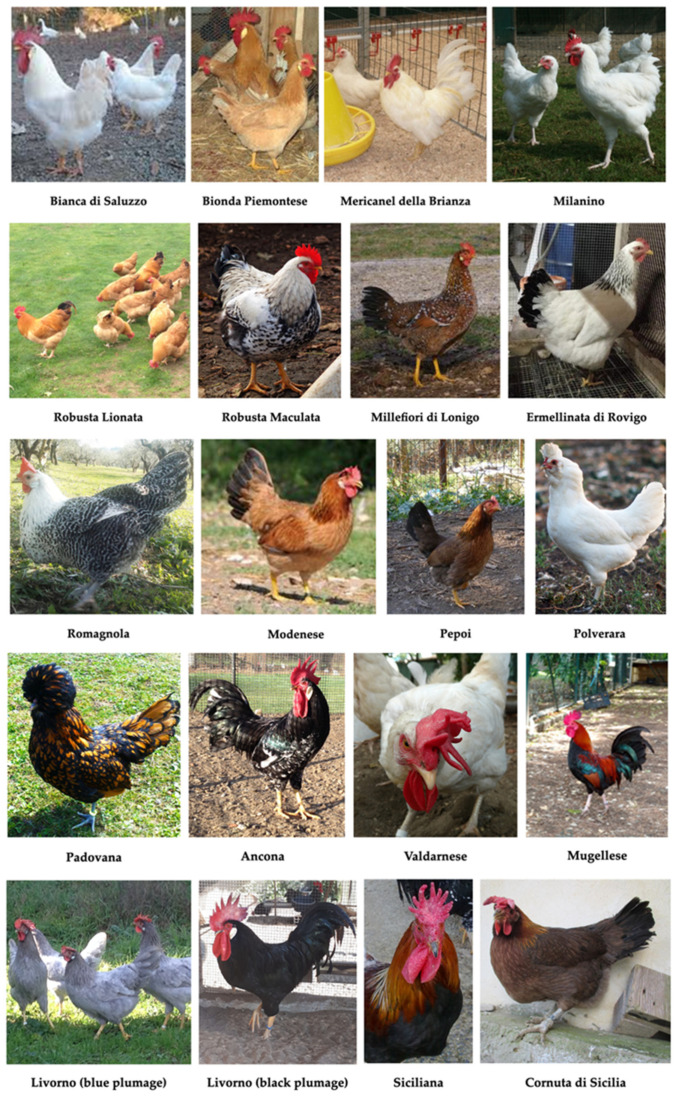
Main native Italian chicken breeds. Reproduced with permission from prof. Silvia Cerolini, TuBAvI Project-coordinator; published at www.pollitaliani.it/en/ (accessed on 6 February 2021) [66].

Table 3 reports the total population sizes for each native Italian chicken breed across the 121 farms surveyed. A total of 15,562 individual birds were recorded, belonging to 21 different native Italian breeds (Figure 2), 18 of which are recognized by the Italian Ministry of Agriculture and admitted for inclusion in the Italian registry of native poultry breeds [10]. Eighty-seven percent of the recorded birds were bred by F, and the remaining 13% by FB.

The largest population of a native breed was observed for the Bionda Piemontese (n = 3400), representing 22% of all native breed chickens (*p* < 0.01), followed by Livorno (n = 1841) and Nostrana di Morozzo (n = 1831). The Bionda Piemontese was the most common native breed reared by F (constituting 24%), significantly greater than the number of birds of this breed reared by FB (4%, *p* < 0.01). The second most common native breed reared by F was Nostrana di Morozzo (13%), followed by Livorno (10%), Polverara (8%), and then all the remaining breeds. The most common native breed to be reared by FB was Livorno (25%, *p* < 0.01), followed by Valdarnese Bianca (17%), Romagnola (11%), then all the remaining breeds to lesser extents. The Bianca di Saluzzo (6%), Ermellinata di Rovigo (6%), Milanino (0.96%), Millefiori di Lonigo (6%), Modenese (0.15%), and Pépoi (7%) were exclusively reared by F. The Cornuta di Sicilia was solely reared by FB (0.91%). Cornuta di Sicilia and Modenese consisted of extremely few individuals (around 20 birds each). With regard to Collo Nudo Italiano, Millefiori Piemontese, Pollo Trentino, and Tirolese breeds, no individuals were identified.

**Figure 3:**
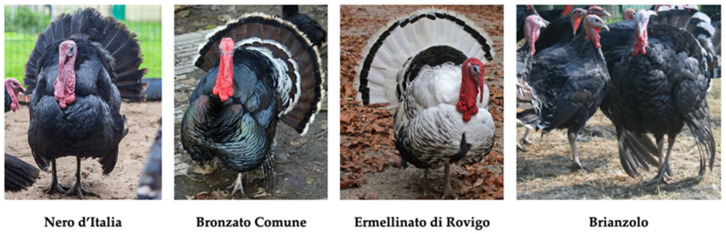
Main native Italian turkey breeds. Reproduced with permission from prof. Silvia Cerolini, TuBAvI Project-coordinator; published at www.pollitaliani.it/en/ (accessed on 6 February 2021) [66].

Seven native Italian turkey breeds were identified as being reared by the breeders of this study, with a total of 1010 individuals (Table 4). The Bronzato Comune (44%, n = 445) and Ermellinato di Rovigo (42%, n = 425) breeds showed the highest population sizes (*p* < 0.01). These two breeds were only kept by F, who showed an evident preference for them over other breeds (Bronzato Comune 49%, Ermellinato di Rovigo 46%; Figure 3). The Parma e Piacenza (0.89%, n = 9) and Brianzolo (1.5%, n = 15; Figure 3) breeds had the smallest population sizes. Bronzato dei Colli Euganei (5%) and Nero d’Italia (3.5%; Figure 3) were reared exclusively by FB, who presented a preference towards the former (53%, *p* < 0.01). Romagnolo turkeys was the only native turkey breed to be bred by both breeder categories, but with significantly higher numbers among FB (10.5%, *p* < 0.01).

### 3.2. Housing and Furnishing

Three types of chicken shed structure were observed: sheds without outdoor access, sheds with outdoor access to an enclosed run, and outdoor pens (Table 5). Overall, breeders preferred chicken sheds with outdoor access to an enclosed run (*p* < 0.01). This trend was also observed for the F breeder category (*p* < 0.01). Among FB, however, outdoor pens were most diffuse (67%, *p* < 0.01). In both breeder categories, chicken sheds without outdoor access were the least common (7%).

#### 3.2.1. Shed and Pen Design According to Breeder Category

Shed characteristics are reported in Table 6. The surface area of most chicken sheds was less than 100 m^2^ (66%, *p* < 0.01). None of the sheds used by FB exceeded a surface area of 100 m^2^. Of the facilities used by F, 60% were less than 100 m^2^, 28% were 100–300 m^2^, and 11% were larger than 300 m^2^. Overall, the majority of sheds used by all breeders were fully closed (59%, *p* < 0.05); the same trend was also seen for F only (65%, *p* < 0.01), but no significant difference was noted for FB (*p* > 0.05). No specific preferences were revealed regarding choice of construction material considering all breeder responses or F alone. The chicken sheds used by FB were most frequently constructed in masonry (54%, *p* < 0.05).

Regarding the use of chicken sheds equipped with vs. without a heating system, no differences were observed between the two possibilities in the responses from all breeders, or when considering the responses from F only (Table S2). A heating system was rarely used by FB (87% did not heat their chicken sheds, *p* < 0.01; Table S2). Levels of ventilation and lighting in the sheds mainly varied according to weather conditions, and extremely few breeders made efforts to measure environmental parameters (temperature, relative humidity (RH), and air quality; Table S2).

The characteristics of enclosed runs and outdoor chicken pens are reported in Table 7. In both breeder categories, most enclosed runs and outdoor pens were bigger than 100 m^2^ (66%, *p* < 0.01) and contained vegetation (84% of all breeders, *p* < 0.01).

Regarding the pen design, the characteristics surveyed regarded whether they were covered, the type of cover used, whether they contained vegetation and if so what kind. The majority of pens in the F category were not covered (69%, *p* < 0.01), whereas the use of a pen cover was more prominent in FB (65%, *p* < 0.05). Canopy fabric (52%) and netting (39%) were the most frequent materials used to cover pens (*p* < 0.01; Table S3). The vegetation inside the pens mainly consisted of trees only (35%) or meadow + bushes + trees (46%, *p* < 0.01). Pens constituting meadow land were mainly polyphyletic (53%) or perennial (35%, *p* < 0.01; Table S3).

#### 3.2.2. Litter and Furnishings

Floor litter was used by all breeders; the different types of litter used are reported in Table 8. Differences were recorded in terms of litter choices between the two breeder categories. The most frequently used litter materials reported considering all responses were wood shavings (30%), straw (23%), and a sand–gravel mixture (19%, *p* < 0.01). Very similar litter choices were reported by F (*p* < 0.05), whereas a strong preference was evident among FB towards wood shavings (47%, *p* < 0.05).

Regarding the management of floor litter, the addition of additives was rarely implemented by breeders (3%, *p* < 0.01). The flip over of the litter was seldom performed by breeders on a whole (16%) or by F (7%, *p* < 0.01). However, this practice was put into effect by 50% of FB (Table S4).

The types of drinkers, feeders and nests used were evaluated and summary data are reported in Table 9. Buckets/makeshift water bowels (42%) and bell drinkers (35%) were the most frequently used types of drinkers (*p* < 0.01). The same drinker type preferences were revealed for F as for all breeder responses (*p* < 0.01). An overall preference was reported by FB was towards buckets/makeshift water bowls (53%, *p* < 0.01). The hopper feeder was the most prevalently used type considering all responses (52%, *p* < 0.01). The distribution of water (70%) and feed (92%) was mostly performed manually (*p* < 0.01; Table S5). Nests were widely used by all breeders (94%, *p* < 0.01), with a preference towards group nests (69%, *p* < 0.01; Table S5) and open nest boxes (68%, *p* < 0.01; Table 9).

### 3.3. Nutrition

Table 10 reports on the use of professional nutritional assistance and feed characteristics. Overall, breeders did not seek professional nutritional assistance (84%, *p* < 0.01). Regarding feed structure, most breeders offered it in the crumb format only (48%, *p* < 0.01). Similarly, F most frequently fed a crumb only feed (52%, *p* < 0.01), whereas the preference of FB was distributed between crumbs (41%), milled–crumb–pellet mixtures (31%) or milled feeds (25%, *p* < 0.01). Regarding the primary feed material, no overall preference was evident for commercial complete diets, self-produced diets, or a combination of the two when considering all breeder responses and F responses only. FB, however, were less likely to produce the feed themselves (12%, *p* < 0.01).

Among the breeders that used homegrown primary materials for producing their own feeds, the most common raw material was maize (88%, *p* < 0.01). The home production of soybean was more frequently performed by FB (53%) than by F (25%, *p* < 0.05; Table S6).

### 3.4. Flock Health and Biosecurity

Variables related to bird health management practices are reported in Table 11. Overall, the majority of breeders recruited the professional assistance of a veterinary (70%, *p* < 0.01). This trend was also evident in the F breeder category (80%, *p* < 0.01), whereas no overriding preference was evident in FB. Daily flock inspections were reported by all breeders. Among F, inspections were mainly performed twice a day (51%, *p* < 0.01), but only once a day by FB (68%, *p* < 0.01).

Data pertaining to flock vaccinations and medical treatments against ectoparasites and endoparasites are reported in Table S7. One hundred percent of flocks were vaccinated against Newcastle Disease. Marek’s Disease vaccination was performed by the majority of breeders (68%, *p* < 0.01). Fowl pox vaccination (70%, *p* < 0.01) and ectoparasite treatments (72%, *p* < 0.01) were also widely performed by the F breeder category. More detailed statistics regarding all the disease vaccinations and medical treatments surveyed are reported in Table S7. Regarding the location of farms, most facilities were situated far from industrial areas (92%, *p* < 0.01) or major roads (82%, *p* < 0.01). The ownership of a cold storage room for dead animals was more common in F (43%) than the FB breeder category (11%, *p* < 0.01; Table S8).

Technical formation related to employees and sanitary procedures adopted are reported in Table 12. Employee training was significantly more frequent among F (76%, *p* < 0.01). Depopulation between one cycle and the next was only performed by 50% of F. Nearly all breeder facilities lacked a vehicle disinfection system (93%, *p* < 0.01).

The measures taken to protect facilities against vermin are reported in Table 13. Anti-bird nets on chicken shed openings were largely used (65% of all breeders, *p* < 0.01). The majority of F also implemented measures to protect against rodent infestations (74%, *p* < 0.01). These practices were applied by approx. half of FB. The most common frequency of interventions taken against rodents in the feed storeroom was once every 30–60 days (43% of all breeders; Table S9).

## 4. Discussion

In many countries, the traits that come to characterize indigenous village chicken breeds are the consequence of centuries of crossbreeding with exotic breeds and random breeding within a flock, making it almost impossible to standardize productive performances and phenotypic/genotypic characteristics [70]. In Italy, breeders choosing to rear local breeds are relatively few in number [63]. Their reason for doing so is most likely due to their passion towards a specific breed. To increase the numbers of these now rare birds and the interest of breeders towards unusual native poultry breeds, producer associations play an important role in promoting awareness about the specific virtues/benefits of traditional poultry products [71].

Numerous different poultry species are reared by rural smallholders around the world. The most common species is the chicken [70,72,73] followed by guinea fowl, ducks, pigeons, turkeys, and geese [70]. This same tendency was observed in the present study, with the exception of pigeons, which were reported to a lesser degree.

According to the FAO, a breed is categorized as “endangered” if the overall population size lies between 1000 and 1200 specimens and is shown to be decreasing, and the percentage of females to males of the same breed is below 80% [1]. Regarding the native Italian breeds surveyed across 121 Italian farms in the present study, encouraging data emerged in relation to the Bionda Piemontese (n = 3400), catalogued as endangered according to the FAO [3]. The FAO also lists the Padovana as endangered; here, 1180 birds were recorded. Another endangered breed according to the FAO is the Bianca di Saluzzo [3]; in this survey, its population status appears to be worse, with only 874 specimens reported.

The most common breed reported in the F breeder category was the Bionda Piemontese (n = 3319), a medium-sized breed [35] formerly considered as dual-purpose, but nowadays mainly used for meat production [17,35]. This result was not unexpected since its geographical place of origin is the Italian region with the third highest concentration of poultry meat farms [63]. The Nostrana di Morozzo, a breed that originates from the Bionda Piemontese, was the second most common breed reared by F breeders (n = 1796). A characteristic of these two breeds is their capacity to produce a highly prized niche product, capons.-The Cappone di San Damiano d’Asti and the Cappone di Morozzo; this latter is listed in the products of the slow-food foundation for biodiversity [74]. In the past, the Bionda Piemontese and the Bianca di Saluzzo were rarely found outside their region of origin, and the Padovana was listed as threatened [4]. Nevertheless, efforts to characterize the genetic heritage of these breeds has been carried out [4], and, as mentioned above, the amount of literature available on these breeds, especially in relation to their genetic characterization, reflects the growing research attention they are receiving (on the Bianca di Saluzzo and Bionda Piemontese, see: [14,15,16,17,18]; on the Padovana, see: [7,19,20,22,23]).

Other breeds listed as “endangered” comprise the Valdarnese Bianca, Romagnola, Mericanel della Brianza, Valdarno Nera, and Modenese [3]. The situation of these breeds, especially the latter three, is serious. The present survey revealed the latter three to make up less than 1% of all native breed chickens surveyed, and the first two make up less than 3% each. In the past, Valdarnese Bianca was already reported as poorly widespread [4], and its risk status continues to be serious (n = 398). The conservation risk status of the Mericanel della Brianza (n = 140) has worsened over the last 20 years [4]. Evidence of some improvements also emerged from this work; for instance, a 2001 investigation detected no individuals of Romagnola, Valdarno Nera, or Modenese, and thus could not exclude the possibility that they had become extinct [4], whereas flock sizes equal to 369, 59, and 20 were detected in the present study, respectively; the situation for these breeds nonetheless remains extremely serious.

A breed’s risk status also seems to correlate with the number of research studies performed on that breed; for example, no manuscripts exist pertaining to Valdarno Nera, and only one publication exists on the phylogeny and genetic relationships of the Modenese breed [11]. This situation highlights the importance of localizing and identifying flocks of the different breeds because in order to perform conservation programs and research projects, up-to-date knowledge about the existence and whereabouts of flocks is essential.

A breed is categorized as “critical” if the overall population size is less than or equal to 120 and decreasing, and the percentage of females being bred to males of the same breed is below 80%. The breeds listed as “critical” by the FAO [3] include the Ancona and the Mugellese, and these breeds each contributed to about 2% of the birds being reared on the farms surveyed. The low population size of the Ancona breed (n = 379) was not expected since this breed is well known and was previously reported to be widespread in Italy [4]. This result could be due to the higher preference observed for the Livorno breed (25%) over the Ancona (9%) as an egg-laying hen, as revealed for FB. The risk status of the Mugellese was shown to have worsened (n = 277) with respect to 20 years ago, when it was a well-known and common breed [4]. The spread of artificial incubators is one reason underlying the decline of these flocks since breeders replaced the Mugellese hens, well-known for their brooding aptitude, and therefore specifically kept for this purpose, with this technology [66]. As reported above, some papers addressing the genetics of the Ancona breed are available [11,12,13]; on the other hand, no genetic surveys were found in relation to the Mugellese. The conservation statuses of Ermellinata di Rovigo and Millefiori di Lonigo were also classified as critical by the FAO [3]. Here, each breed made up approx. 5% of all native breed specimens kept by the breeders surveyed. In the abovementioned 2001 survey, Ermellinata di Rovigo was widely diffuse, whereas no individuals of Millefiori di Lonigo were detected, which was thus reported to be extinct [4]. Therefore, we can report that the risk status of Ermellinata di Rovigo has likely worsened (n = 828), whilst some improvement seemed to have been achieved in relation to Millefiori di Lonigo (n = 755). Regarding the publication of genetic studies, some data is available for Ermellinata di Rovigo [7,19,20,22,23], whereas only one publication was identified in relation to Millefiori di Lonigo [19].

Regarding chicken breed preferences in the FB category, the most common bird was an egg-laying breed, the Livorno (n = 501). In contexts of backyard poultry production, families mainly keep hens for self-consumption [75,76,77,78]. In Italy, the choice of the Livorno as an egg-laying hen is linked to this breed’s high egg production capacity, which can readily meet a family’s consumption needs and provide potential extra income through the selling of sought-after eggs. Owners of backyard chickens in the USA also demonstrate a preference towards egg-laying breeds, with egg color also being a matter that affects breed choice [72]. The Livorno and the Polverara are reported as being at “critical” risk of extinction according to the FAO [3]. Nonetheless, the Livorno was the second most reared chicken breed across all breeders. That said, considering that the Livorno is one of the most well-known native Italian chicken breeds, we had actually expected to observe a larger total population size for this breed, also because its diffusion was very widespread in the past [4]. Different plumage color varieties of the Livorno breed exist. Thus, ascertaining the flock sizes of the different varieties will be important so that the appropriate interventions can be put into place to safeguard the varieties more at risk. In fact, for some color varieties, the risk status might be highly endangered. An additional aspect to highlight regards the White Livorno, which is often confused with the White Leghorn by nonexperts, and to which the former is unrelated. As mentioned above, several genetic studies have been published in the past 10 years in relation to the Livorno breed [11,12,13,15,25].

Concerning the risk status of the Polverara, this breed was previously determined to be threatened, but projects have since been carried out to try to safeguard the breed [4]. Indeed, some improvements were achieved, and the present study showed the Polverara to constitute 7% of all native breed chickens kept on the 121 farms surveyed (n = 1093). Genetic data about this breed have also been obtained [7,19,20].

Another risk status listed by the FAO [1] is the “critical-maintained”. This refers to breed populations for which active conservation programs are in place or are being maintained by commercial companies or research institutions. This status has been applied to Pépoi and Robusta Lionata [3]. In the past, the Pépoi was widely diffuse across Italy, whereas a poor distribution was reported for Robusta Lionata [4]. In this study, 6% of all chickens belonged to the Pépoi breed (n = 899), whereas only 3% belonged to Robusta Lionata (n = 452). Thus, we can propose the risk status of Pépoi to have worsened, whereas the poor status of Robusta Lionata has simply persisted. Reports on the genetic characteristics of both breeds are available (for Pepoi, see: [7,19,20,22,23,24]; for Robusta Lionata, see [7,19,22,24]).

The risk status “endangered-maintained” is applied to endangered populations for which active conservation programs are in place, or populations are being maintained by commercial companies or research institutions [1]. Robusta Maculata is one breed classified as such [3]. Its status was not any better in the past [4]. In this study, 433 individuals were identified, and several genetic studies have also addressed the Robusta Maculata breed over the last 12 years [19,20,22,24].

The Siciliana is classified as “vulnerable” [3]. Twenty years ago, its risk status indicated it to be poorly diffuse [4]. Just 186 individuals were detected in the present study, an exceedingly worrying datum. Concerning the genetic aspects of this breed, just one study is available in the literature [15].

No reference is made to Milanino, Nostrana di Morozzo, or Cornuta di Sicilia in the FAO database [3], neither are they listed in the Registry of Native Poultry Breeds by the MIPAAF [10]. Additionally, no research studies have been published in relation to either of the last two breeds, whereas Zanon and Sabbioni reported no individuals of Milanino in their 2001 survey [4]. Some improvements have since been made with regard to the Milanino: at least 1% of all chickens kept by all breeders belonged to this breed (n = 130); only limited data is available about their genetic features [15]. Here, we show that 12% of all chickens belonged to the Nostrana di Morozzo (n = 1831), i.e., the same proportion as the Livorno breed. No individuals were identified for the breeds: Collo Nudo Italiano, Millefiori Piemontese, Pollo Trentino, and the Tirolese breeds.

Regarding turkey breeds, the FAO reported Bronzato Comune and Ermellinato di Rovigo as “critical maintained” [3]. In the past, Bronzato Comune was widely diffuse in Italy whilst Ermellinato di Rovigo was poorly represented [4]. In this study, breeders showed a high level of preference for both these breeds: 44% (n = 445) and 42% (n = 425) of turkeys recorded were of these breeds, respectively. The risk status of the Bronzato Comune has thus remained constant over time considering the 121 breeding facilities surveyed, whereas an improvement can be observed in relation to Ermellinato. Some genetic information is available on both breeds [24,28].

Another turkey breed reported as “endangered-maintained” by FAO [3] is the Castano Precoce. In the past, its status was listed as threatened, but some efforts were carried out to augment the flocks of this breed [4]. Nevertheless, in the present survey, no individuals were detected, so its risk status has yet to be ascertained, and the possibility remains that it may have worsened.

The Bronzato dei Colli Euganei turkey breed was previously reported to be threatened, and efforts were being made to obtain genetic data about this bird [4]. As shown in the present study, despite 5% of turkeys reported belonging to this breed (n = 50), it is certainly still under threat of extinction. Little information is available regarding its genetic features [29].

Brianzolo turkeys were recognized as threatened 20 years ago [4], and the data of this present study do not suggest any change to this risk status, with less than 2% of the turkeys identified belonging to the breed (n = 15). Some genetic information about this breed has been published [28,29]. Parma e Piacenza and Romagnolo turkey breeds were previously classified as extinct [4]. At present, 1% of the turkeys kept belonged to Parma e Piacenza (n = 9) and 3% belonged to Romagnolo (n = 31) turkeys: an improvement, but the risk status of these breeds remains serious.

Regarding the demographic data of Italian poultry breeders, the majority are men, aged 30–70 years, and perform this activity as a secondary job or hobby, reflecting their passion for one or more poultry breeds. These data lie in contrast with the situation in developing countries, where poultry keeping is a traditionally performed by women, providing an additional means of livelihood for their families [70,73,79]. Moreover, the flock composition in developing countries depends on the goals of the poultry farm, and in certain cases it depends on the phenotypic characteristics of the birds; for example, the preference for a specific plumage color, which renders birds less visible to predators [70]. The choices of Italian breeders are mainly linked to the breed’s geographical origins and specific phenotypic or productive characteristics [80].

As evidenced by the kind of sheds provided by breeders, especially FB, a good level of awareness towards the birds was observed. Birds were provided with outdoor runs including vegetation, and were thus able to scratch, forage, dustbathe, and sun themselves. This finding is in accordance with those of other authors [72,81,82]. Nevertheless, a problem often faced by breeders offering outdoor areas regards the risk of attack by predators; as a result, night-time confinement was widely adopted [72,73,76,77]. In this study, and in accordance with other authors [72], especially fancy breeders also reported their use of measures to avoid problems with predators during the day. The most common measure taken involved the overhead covering of outdoor spaces despite the associated expenses entailed. Another aspect suggesting that breeders invest in their flocks’ security regards the kind of sheds used, with breeders preferring masonry structures to improvised structures. This contrasts highly with village households in developing countries, where chickens are generally kept inside their owners’ houses [70].

Regarding litter materials, almost 70% of breeders preferred those of organic origin. This agrees with the findings of some authors [72,83], but contrasts with those of others [78,80] who report a preference towards inorganic material. Litter material choice is usually linked to factors such as availability, cost, and allowance for cleaning and ventilation [70]. When performing the cleaning procedures, the use of an organic material as litter is certainly lighter, thus easier to lift and compost, making it a practice that can be performed more often, especially considering that chiefly in the F category, the litter was seldom flipped over.

Water was predominantly provided using simple or improvised equipment (i.e., buckets or makeshift water bowls), although specific attention was given to the provision of clean and fresh water. Certainly, the source of water is more important than how it is offered. Fresh water sources are generally easily obtained in Europe, in contrast with developing countries, where fetching and carrying water constitutes a crucial and labor-intensive task [70,79].

In general, no preference was observed for a specific feed source; only FB manifested a specific lack of preference towards a grain-based homemade feed. Other authors report backyard poultry raisers to have a high preference for a mixed ration of commercial feed and kitchen scraps [72], or scavenged household leftovers plus insects, fruit and vegetable crops, grass, grain, and various supplementary feedstuffs [73,77].

As expected, and in agreement with previous reports [73], particular attention was paid by breeders to egg collection practices, with a high percentage of breeders offering nests to minimize the chance of eggs being laid on the floor [70].

Concern for the maintenance of healthy flocks was demonstrated by the common practice of vaccination and the recruitment of professional veterinarian support, especially in the F breeder category. Furthermore, this latter category was largely aware of the risks of disease transmission from wild birds and the importance of the correct disposal of dead birds. This finding contrasts with those of other studies [81,84,85,86]. Nevertheless, a lack of knowledge about biosecurity practices was observed as very few breeders employed a vehicle disinfection system, and depopulation between cycles was only put into practice by half of F breeders.

## 5. Conclusions

Analysis of data gathered from 121 native Italian poultry breeders reveal low population sizes of all native Italian poultry breeds. Only four breeds presented population sizes that exceeded 1000 individuals each, all other breeds, including turkey breeds, were much smaller. This means that the conservation risk statuses of all breeds are a matter of great concern, with all at risk of becoming endangered—some more so than others.

In general, the responses from breeders show that they are aware and care about the needs of birds. The role of breeders is central to maintaining the Italian bird genetic pool. Additional programs involving breeders, researchers, and public entities should be developed, existing projects should continue, and all of the above should work together towards the shared goal that is the preservation of native Italian poultry breeds. Additionally, active communication is required to share information about specific breeds as much as possible, and to promote their virtues and valorize their products as well as to facilitate access to these breeds, since the geographic distribution of each breed is often linked to their territory of origin.

## Figures and Tables

**Table 1:** Personal information: all breeders surveyed and divided according to breeder category.

	All Breeders		Farmers		Fancy Breeders		χ^2^ ^1^
Variable	n	%	n	%	n	%	
Survey response	121	100	75	62	46	38	**
Gender	(n = 120)		(n = 74)		(n = 46)		
Male	92 ^A^	76.67	50 ^A^	67.57	42 ^A^	91.30	**
Female	28 ^B^	23.33	24 ^B^	32.43	4 ^B^	8.70	**
Age of male breeders	(n = 81)		(n = 48)		(n = 33)		
<than 30 years old	12 ^B^	14.81	5 ^C^	10.42	7	21.21	NS
30–50 years old	29 ^A^	35.80	15 ^B^	31.25	14	42.42	NS
51–70 years old	33 ^A^	40.74	26 ^A^	54.17	7	21.21	**
>than 70 years old	7 ^B^	8.64	2 ^C^	4.17	5	15.15	NS
Age of female breeders	(n = 23)		(n = 19)		(n = 4)		
<than 30 years old	0 ^B^	0.00	0 ^C^	0.00	0	0.00	-
30–50 years old	9 ^A^	39.13	6 ^A,B^	31.58	3	75.00	NS
51–70 years old	13 ^A^	56.52	12 ^A^	63.16	1	25.00	NS
>than 70 years old	1 ^B^	4.35	1 ^C,B^	5.26	0	0.00	NS
Main vs. secondary occupation	(n = 118)		(n = 74)		(n = 44)		
Main	27 ^B^	22.88	24 ^B^	32.43	3 ^B^	6.82	**
Secondary	91 ^A^	77.12	50 ^A^	67.57	41 ^A^	93.18	**

^1^ Chi square test for a single variable between the two breeder categories, i.e., within row comparisons; significance levels: ** *p* < 0.01. ^A–C^ Observations with different superscripts within the column are significantly different (χ^2^-test *p* < 0.01).

**Table 2:** Number of farms rearing chickens only or chickens plus other bird species: summary data for all breeders surveyed and divided according to breeder category.

	All Breeders		Farmers		Fancy Breeders		χ^2^ ^1^
Variable	n	%	n	%	n	%	
Bird species	(n = 121)		(n = 75)		(n = 46)		
Chickens	69 ^a^	57.02	41	54.67	28 ^a^	60.87	NS
Chickens + other bird sp.	52 ^b^	42.98	34	45.33	18 ^b^	39.13	NS
Other species reared	(n = 52)		(n = 34)		(n = 18)		
Turkeys	30 ^A^	57.69	16 ^A^	47.06	14 ^A^	77.78	*
Ducks	23 ^A^	44.23	14 ^A^	41.18	9 ^A,B^	50.00	NS
Geese	22 ^A^	42.31	15 ^A^	44.12	7 ^B^	38.89	NS
Guinea fowl	22 ^A^	42.31	14 ^A^	41.18	8 ^A,B^	44.44	NS
Pigeons	4 ^B^	7.69	1 ^B^	2.94	3 ^B,C^	16.67	NS
Peacocks	4 ^B^	7.69	3 ^B^	8.82	1 ^C^	5.56	NS
Quails	4 ^B^	7.69	3 ^B^	8.82	1 ^C^	5.56	NS
Pheasants	2 ^B^	3.85	1 ^B^	2.94	1 ^C^	5.56	NS
Partridges	1 ^B^	1.92	0 ^B^	0.00	1 ^C^	5.56	NS

^1^ Chi square test for a single variable between the two breeder categories, i.e., within row comparisons; significance levels: * *p* < 0.05; NS, nonsignificant (*p* > 0.05). ^A–C^ Observations with different superscripts within the column are significantly different (χ^2^-test *p* < 0.01). ^a,b^ Observations with different superscripts within the column are significantly different (χ^2^-test, *p* < 0.05).

**Table 3:** Native Italian chicken breed population sizes: summary data for all breeders and divided according to breeder category.

	All Breeders		Farmers		Fancy Breeders		χ^2^ ^1^
Variable	n	%	n	%	n	%	
Italian Chicken Breed	(n = 15,562)		(n = 13,588)		(n = 1974)		
Ancona	379 ^G,H^	2.44	208 ^I^	1.53	171 ^D,E^	8.66	**
Bianca di Saluzzo	874 ^D^	5.62	874 ^E,F^	6.43	0 ^J^	0.00	**
Bionda Piemontese	3400 ^A^	21.85	3319 ^A^	24.43	81 ^F^	4.10	**
Collo Nudo Italiana	-	-	-	-	-	-	
Ermellinata di Rovigo	828 ^D,E^	5.32	828 ^F,G^	6.09	0 ^J^	0.00	**
Livorno	1841 ^B^	11.83	1340 ^C^	9.86	501 ^A^	25.38	**
Mericanel della Brianza	140 ^K^	0.90	131 ^J^	0.96	9 ^H,I^	0.46	**
Millefiori di Lonigo	755 ^E^	4.85	755 ^G^	5.56	0^J^	0.00	**
Millefiori Piemontese	-	-	-	-	-	-	
Modenese	20 ^M^	0.13	20 ^M^	0.15	0 ^J^	0.00	**
Mugellese	277 ^I^	1.78	92 ^K^	0.68	185 ^D^	9.37	**
Padovana	1180 ^C^	7.58	952 ^E^	7.01	228 ^C^	11.55	**
Pépoi	899 ^D^	5.78	899 ^E,F^	6.62	0 ^J^	0.00	**
Pollo Trentino	-	-	-	-	-	-	
Polverara	1093 ^C^	7.02	1090 ^D^	8.02	3 ^I,J^	0.15	**
Robusta Lionata	452 ^F^	2.90	444 ^H^	3.27	8 ^H,I^	0.41	**
Robusta Maculata	433 ^F,G^	2.78	419 ^H^	3.08	14 ^H^	0.71	**
Romagnola	369 ^H^	2.37	149 ^J^	1.10	220 ^C^	11.14	**
Siciliana	186 ^J^	1.20	41 ^L^	0.30	145 ^E^	7.35	**
Valdarnese Bianca	398 ^F,G,H^	2.56	57 ^L^	0.42	341 ^B^	17.27	**
Valdarno Nera	59 ^L^	0.38	44 ^L^	0.32	15 ^H^	0.76	**
Tirolese o Tirolerhuhn	-	-	-	-	-	-	
Other local bird populations ^2^							
Cornuta di Sicilia	18 ^M^	0.12	0 ^N^	0.00	18 ^H^	0.91	**
Milanino	130 ^K^	0.84	130 ^J^	0.96	0 ^J^	0.00	**
Nostrana di Morozzo	1831 ^B^	11.77	1796 ^B^	13.22	35 ^G^	1.77	**

^1^ Chi square test for a single variable between the two breeder categories, i.e., within row comparisons; significance levels: ** *p* < 0.01. ^A–N^ Observations with different superscripts within the column are significantly different (χ^2^-test *p* < 0.01). ^2^ Breeds not recognized by the Italian Ministry for Agricultural Policies.

**Table 4:** Native Italian turkey breed population sizes: summary data for all breeders and divided according to breeder category.

	All Breeders		Farmers		Fancy Breeders		χ^2^ ^1^
Variable	n	%	n	%	n	%	
Italian turkey breed	(n = 1010)		(n = 915)		(n = 95)		
Brianzolo	15 ^D^	1.49	15 ^B^	1.64	0 ^D^	0.00	**
Bronzato Comune	445 ^A^	44.06	445 ^A^	48.63	0 ^D^	0.00	**
Bronzato dei Colli Euganei	50 ^B^	4.95	0 ^C^	0.00	50 ^A^	52.63	**
Castano Precoce	-	-	-	-	-	-	
Ermellinato di Rovigo	425 ^A^	42.08	425 ^A^	46.45	0 ^D^	0.00	**
Nero d’Italia	35 ^BC^	3.47	0 ^C^	0.00	35 ^B^	36.84	**
Parma e Piacenza	9 ^D^	0.89	9 ^B^	0.98	0 ^D^	0.00	**
Romagnolo	31 ^C^	3.07	21 ^B^	2.30	10 ^C^	10.53	**

^1^ Chi square test for a single variable between the two breeder categories, i.e., within row comparisons; significance levels: ** *p* < 0.01. ^A–D^ Observations with different superscripts within the column are significantly different (χ^2^-test *p* < 0.01).

**Table 5:** Types of housing structures used: responses from all breeders and divided according to breeder category.

	All Breeders		Farmers		Fancy Breeders		χ^2^ ^1^
Variable	n	%	n	%	n	%	
Housing structures	(n = 121)		(n = 75)		(n = 46)		
Shed	9 ^C^	7.44	7 ^B^	9.33	2 ^C^	4.35	NS
Shed and enclosed run	68 ^A^	56.20	55 ^A^	73.33	13 ^B^	28.26	**
Outdoor pens	44 ^B^	36.36	13 ^B^	17.33	31 ^A^	67.39	**

^1^ Chi square test for a single variable between the two breeder categories, i.e., within row comparisons; significance levels: ** *p* < 0.01; NS, nonsignificant (*p* > 0.05). ^A–C^ Observations with different superscripts within the column are significantly different (χ^2^-test *p* < 0.01).

**Table 6:** Chicken shed design: responses from all breeders and divided according to breeder category.

	All Breeders		Farmers		Fancy Breeders		χ^2^ ^1^
Variable	n	%	n	%	n	%	
Shed surface area (m^2^)	(n = 62)		(n = 53)		(n = 9)		
<than 100 m^2^	41 ^A^	66.13	32 ^A^	60.38	9 ^A^	100.00	NS
100–300 m^2^	15 ^B^	24.19	15 ^B^	28.30	0 ^B^	0.00	NS
>than 300 m^2^	6 ^B^	9.68	6 ^B^	11.32	0 ^B^	0.00	NS
Types of sheds	(n = 75)		(n = 60)		(n = 15)		
Fully closed sheds	44 ^a^	58.67	39 ^A^	65.00	5	33.33	*
Open sheds	31 ^b^	41.33	21 ^B^	35.00	10	66.67	*
Construction materials	(n = 74)		(n = 61)		(n = 13)		
Masonry	30	40.54	23	37.70	7 ^a^	53.85	NS
Prefabricated	19	25.67	18	29.51	1 ^b^	7.69	NS
Wood	25	33.79	20	32.79	5 ^a,b^	38.46	NS

^1^ Chi square test for a single variable between the two breeder categories, i.e., within row comparisons; significance levels: NS, nonsignificant (*p* > 0.05). ^A–C^ Observations with different superscripts within the column are significantly different (χ^2^-test *p* < 0.01). ^a,b^ Observations with different superscripts within the column are significantly different (χ^2^-test, *p* < 0.05).

**Table 7:** Enclosed run and outdoor pen design: responses from all breeders and divided according to breeder category.

	All Breeders		Farmers		Fancy Breeders		χ^2^ ^1^
Variable	n	%	n	%	n	%	
Dimensions (m^2^)	(n = 96)		(n = 63)		(n = 32)		
<than 50 m^2^	23 ^B^	23.96	12 ^B^	19.05	11 ^A^	34.38	NS
50–100 m^2^	9 ^C^	9.38	6 ^B^	9.52	3 ^B^	9.37	NS
>than 100 m^2^	63 ^A^	65.63	45 ^A^	71.43	18 ^A^	56.25	NS
Vegetation	(n = 103)		(n = 68)		(n = 35)		
Yes	87 ^A^	84.47	59 ^A^	87.76	28 ^A^	80.00	NS
No	16 ^B^	15.53	9 ^B^	13.24	7 ^B^	20.00	NS

^1^ Chi square test for a single variable between the two breeder categories, i.e., within row comparisons; significance levels: NS, nonsignificant (*p* > 0.05). ^A–C^ Observations with different superscripts within the column are significantly different (χ^2^-test *p* < 0.01).

**Table 8:** Use and type of floor litter: responses from all breeders and divided according to breeder category.

	All Breeders		Farmers		Fancy Breeders		χ^2^ ^1^
Variable	n	%	n	%	n	%	
Litter	(n = 77)		(n = 62)		(n = 15)		
Yes	77 ^A^	100.00	62 ^A^	100.00	15 ^A^	100.00	NS
No	0 ^B^	0.00	0 ^B^	0.00	0 ^B^	0.00	-
Type of litter	(n = 77)		(n = 62)		(n = 15)		
Straw	18 ^A,B^	23.38	16 ^a^	25.81	2 ^b^	13.33	NS
Wood shavings	23 ^A^	29.87	16 ^a^	25.81	7 ^a^	46.67	NS
Rice lulls	11 ^B,C^	14.29	9 ^a,b^	14.52	2 ^b^	13.33	NS
Sand	6 ^C^	7.79	4 ^b^	6.45	2 ^b^	13.33	NS
Gravel	4 ^C^	5.19	3 ^b^	4.84	1 ^b^	6.67	NS
Sand/gravel mixture	15 ^A,B^	19.48	14 ^a^	22.58	1 ^b^	6.67	NS

^1^ Chi square test for a single variable between the two breeder categories, i.e., within row comparisons; significance levels: NS, nonsignificant (*p* > 0.05). ^A–C^ Observations with different superscripts within the column are significantly different (χ^2^-test *p* < 0.01). ^a,b^ Observations with different superscripts within the column are significantly different (χ^2^-test, *p* < 0.05).

**Table 9:** Shed types and pen furnishings: responses from all breeders and divided according to breeder category.

	All Breeders		Farmers		Fancy Breeders		χ^2^ ^1^
Variable	n	%	n	%	n	%	
Drinkers	(n = 110)		(n = 74)		(n = 36)		
Buckets/makeshift water bowls	46 ^A^	41.82	27 ^A^	36.49	19 ^A^	52.78	NS
Troughs	3 ^C^	2.73	2 ^C^	2.70	1 ^C^	2.78	NS
Bell drinkers	39 ^A^	35.45	30 ^A^	40.54	9 ^B^	25.00	NS
Nipples	8 ^B,C^	7.27	3 ^C^	4.05	5 ^B,C^	13.89	NS
A combination of the above	14 ^B^	12.73	12 ^B^	16.22	2 ^C^	5.56	NS
Feeders	(n = 117)		(n = 75)		(n = 42)		
Bowls or pans	19 ^B^	16.24	14 ^B^	18.67	5 ^B^	11.90	NS
Troughs	16 ^B^	13.68	11 ^B^	14.67	5 ^B^	11.90	NS
Hoppers	61 ^A^	52.14	37 ^A^	49.33	24 ^A^	57.14	NS
Others	2 ^C^	1.71	1 ^C^	1.33	1 ^B^	2.38	NS
A combination of the above	19 ^B^	16.24	12 ^B^	16.00	7 ^B^	16.67	NS
Nests	(n = 105)		(n = 66)		(n = 39)		
Open nest box	72 ^A^	68.57	48 ^A^	72.73	24 ^A^	61.54	NS
Closed nest box with litter or metal net	23 ^B^	21.90	12 ^B^	18.18	11 ^B^	28.21	NS
Rollaway nest box with plastic trays	2 ^C^	1.90	2 ^C^	3.03	0 ^C^	0.00	NS
A combination of the above	8 ^C^	7.62	4 ^C^	6.06	4 ^B,C^	10.26	NS

^1^ Chi square test for a single variable between the two breeder categories, i.e., within row comparisons; significance levels: NS, nonsignificant (*p* > 0.05). ^A–C^ Observations with different superscripts within the column are significantly different (χ^2^-test *p* < 0.01).

**Table 10:** Professional nutrition assistance, feed structures and feed sources: responses from all breeders and divided according to breeder category.

	All Breeders		Farmers		Fancy Breeders		χ^2^ ^1^
Variable	n	%	n	%	n	%	
Nutritionist	(n = 97)		(n = 71)		(n = 26)		
Yes	16 ^B^	16.49	15 ^B^	21.13	1 ^B^	3.85	*
No	81 ^A^	83.51	56 ^A^	78.87	25 ^A^	96.15	*
Feed structure	(n = 90)		(n = 58)		(n = 32)		
Milled	21 ^B^	23.33	13 ^B^	22.41	8 ^A^	25.00	NS
Crumbs	43 ^A^	47.78	30 ^A^	51.72	13 ^A^	40.63	NS
Pellets	2 ^C^	2.22	1 ^C^	1.72	1 ^B^	3.13	NS
A combination of the above	24 ^B^	26.67	14 ^B^	24.14	10 ^A^	31.25	NS
Feed sources	(n = 114)		(n = 73)		(n = 41)		
Complete commercial diet	40	35.09	18	24.66	22 ^A^	53.66	**
Self-produced	30	26.32	25	34.25	5 ^B^	12.20	*
Both	44	38.60	30	41.10	14 ^A^	34.15	NS

^1^ Chi square test for a single variable between the two breeder categories, i.e., within row comparisons; significance levels: ** *p* < 0.01; * *p* < 0.05; NS, nonsignificant (*p* > 0.05). ^A–C^ Observations with different superscripts within the column are significantly different (χ^2^-test *p* < 0.01).

**Table 11:** Flock health management: responses from all breeders and divided according to breeder category.

	All Breeders		Farmers		Fancy Breeders		χ^2^ ^1^
Variable	n	%	n	%	n	%	
Veterinarian	(n = 97)		(n = 70)		(n = 27)		
Yes	68 ^A^	70.10	56 ^A^	80.00	12	44.44	**
No	29 ^B^	29.90	14 ^B^	20.00	15	55.56	**
Bird inspection/day (n)	(n = 70)		(n = 51)		(n = 19)		
1×	28 ^A^	40.00	15 ^B^	29.41	13 ^A^	68.42	**
2×	29 ^A^	41.43	26 ^A^	50.98	3 ^B^	15.79	**
>than 2×	13 ^B^	18.57	10 ^B^	19.61	3 ^B^	15.79	NS

^1^ Chi square test for a single variable between the two breeder categories, i.e., within row comparisons; significance levels: ** *p* < 0.01; NS, nonsignificant (*p* > 0.05). ^A,B^ Observations with different superscripts within the column are significantly different (χ^2^-test *p* < 0.01).

**Table 12:** Professional training and biosecurity practices employed: responses from all breeders and divided according to breeder category.

	All Breeders		Farmers		Fancy Breeders		χ^2^ ^1^
Variable	n	%	n	%	n	%	
Employee training	(n = 98)		(n = 71)		(n = 27)		
Yes	60 ^A^	61.22	54 ^A^	76.06	6 ^B^	22.22	**
No	38 ^B^	38.78	17 ^B^	23.94	21 ^A^	77.78	**
Depopulation between cycles	(n = 67)		(n = 56)		(n = 11)		
Yes	28	41.79	28	50.00	0 ^B^	0.00	**
No	39	58.21	28	50.00	11 ^A^	100.00	**
Vehicle disinfection	(n = 108)		(n = 74)		(n = 34)		
Yes	8 ^B^	7.41	8 ^B^	10.81	0 ^B^	0.00	*
No	100 ^A^	92.59	66 ^A^	89.19	34 ^A^	100.00	*

^1^ Chi square test for a single variable between the two breeder categories, i.e., within row comparisons; significance levels: ** *p* < 0.01; * *p* < 0.05. ^A,B^ Observations with different superscripts within the column are significantly different (χ^2^-test *p* < 0.01).

**Table 13:** Vermin control measures implemented: responses from all breeders and divided according to breeder category.

	All Breeders		Farmers		Fancy Breeders		χ^2^ ^1^
Variable	n	%	n	%	n	%	
Anti-bird nets on shed openings	(n = 77)		(n = 62)		(n = 15)		
Yes	50 ^A^	64.94	41 ^A^	66.13	9	60.00	NS
No	27 ^B^	35.06	21 ^B^	33.87	6	40.00	NS
Rodent control in the feed storeroom	(n = 109)		(n = 74)		(n = 35)		
Yes	73 ^A^	66.97	55 ^A^	74.32	18	51.43	*
No	36 ^B^	33.03	19 ^B^	25.68	17	48.57	*
Rodent control within the shed	(n = 110)		(n = 74)		(n = 36)		
Yes	71 ^A^	64.55	54 ^A^	72.97	17	47.22	**
No	39 ^B^	35.45	20 ^B^	27.03	19	52.78	**

^1^ Chi square test for a single variable between the two breeder categories, i.e., within row comparisons; significance levels: ** *p* < 0.01; * *p* < 0.05; NS, nonsignificant (*p* > 0.05). ^A,B^ Observations with different superscripts within the column are significantly different (χ^2^-test *p* < 0.01).

## Data Availability

The data presented in this study are available on request from the corresponding author.

## Supplementary Materials

The following are available online at https://www.mdpi.com/2076-2615/11/2/490/s1, Table S1: Manpower involved in the care and management of flocks: responses from all breeders and divided according to breeder category, Table S2: Environmental housing conditions adopted: responses from all breeders and divided according to breeder category, Table S3: Pen cover and ground vegetation: responses from all breeders and divided according to breeder category, Table S4: Litter management: responses from all breeders and divided according to breeder category, Table S5: Pen furnishings: responses from all breeders and divided according to breeder category, Table S6: Self-production of feed primary materials: responses from all breeders and divided according to breeder category, Table S7: Flock vaccinations and medical treatments performed: responses from all breeders and divided according to breeder category, Table S8: Farm location and presence of a cold storage room for dead animals: responses from all breeders and divided according to breeder category, Table S9: Frequency of interventions against rodents: responses from all breeders and divided according to breeder category.

## References

[1] Food and Agriculture Organization of the United Nations (FAO) (2007). The State of the World’s Animal Genetic Resources for Food and Agriculture; Chief, Electronic Publishing Policy and Support Branch Communication Division.

[2] Directorate-General for Agriculture and Rural Development (2020). Preparatory Action EU Plant and Animal Genetic Resources—Executive Summary.

[3] Food and Agriculture Organization of the United Nations (FAO) (2020). Domestic Animal Diversity Information System (DAD-IS).

[4] Zanon A., Sabbioni A. (2001). Identificazione e salvaguardia genetica delle razze avicole Italiane. Ann. Med. Vet..

[5] Mosca F., Zaniboni L., Stella S., Kuster C.A., Iaffaldano N., Cerolini S. (2018). Slaughter performance and meat quality of Milanino chickens reared according to a specific free-range program. Poult. Sci..

[6] Rizzi C., Marangon A. (2012). Quality of organic eggs of hybrid and Italian breed hens. Poult. Sci..

[7] Zanetti E., De Marchi M., Dalvit C., Cassandro M. (2010). Genetic characterization of local Italian breeds of chickens undergoing in situ conservation. Poult. Sci..

[8] De Marchi M., Dalvit C., Targhetta C., Cassandro M. (2006). Assessing genetic diversity in indigenous Veneto chicken breeds using AFLP markers. Anim. Genet..

[9] Sabbioni A., Zanon A., Beretti V., Superchi P., Zambini E.M. Carcass yield and meat quality parameters of two Italian autochthonous chicken breeds reared outdoor: Modenese and Romagnolo. Proceedings of the WPSA XII European Poultry Conference.

[10] MIPAAF (2014). Disciplinare del Registro Anagrafico Degli Avicoli Autoctoni.

[11] Ceccobelli S., Di Lorenzo P., Lancioni H., Ibanez L.V.M., Tejedor M.T., Castellini C., Landi V., Martinez A.M., Bermejo J.V.D., Pla J.L.V. (2015). Genetic diversity and phylogeographic structure of sixteen Mediterranean chicken breeds assessed with microsatellites and mitochondrial DNA. Livest. Sci..

[12] Ceccobelli S., Di Lorenzo P., Lancioni H., Castellini C., Ibanez L.V.M., Sabbioni A., Sarti F.M., Weigend S., Lasagna E. (2013). Phylogeny, genetic relationships and population structure of five Italian local chicken breeds. Ital. J. Anim. Sci..

[13] Bianchi M., Ceccobelli S., Landi V., Di Lorenzo P., Lasagna E., Ciocchetti M., Sahin E., Mugnai C., Panella F., Sarti F.M. (2011). A microsatellites-based survey on the genetic structure of two Italian local chicken breeds. Ital. J. Anim. Sci..

[14] Soglia D., Sacchi P., Sartore S., Maione S., Schiavone A., De Marco M., Bottero M.T., Dalmasso A., Pattono D., Rasero R. (2017). Distinguishing industrial meat from that of indigenous chickens with molecular markers. Poult. Sci..

[15] Strillacci M.G., Cozzi M.C., Gorla E., Mosca F., Schiavini F., Roman-Ponce S.I., Lopez F.J.R., Schiavone A., Marzoni M., Cerolini S. (2017). Genomic and genetic variability of six chicken populations using single nucleotide polymorphism and copy number variants as markers. Animal.

[16] Sartore S., Sacchi P., Soglia D., Maione S., Schiavone A., De Marco M., Ceccobelli S., Lasagna E., Rasero R. (2016). Genetic variability of two Italian indigenous chicken breeds inferred from microsatellite marker analysis. Br. Poult. Sci..

[17] Sartore S., Soglia D., Maione S., Sacchi P., De Marco M., Schiavone A., Sponza S., Dalmasso A., Bottero M.T., Pattono D. (2014). Genetic traceability of two local chicken populations, Bianca di Saluzzo and Bionda Piemontese, versus some current commercial lines. Ital. J. Agron..

[18] De Marco M., Miro S.M., Tarantola M., Bergagna S., Mellia E., Gennero M.S., Schiavone A. (2013). Effect of genotype and transport on tonic immobility and heterophil/lymphocyte ratio in two local Italian breeds and Isa Brown hens kept under free-range conditions. Ital. J. Anim. Sci..

[19] Viale E., Zanetti E., Ozdemir D., Broccanello C., Dalmasso A., De Marchi M., Cassandro M. (2017). Development and validation of a novel SNP panel for the genetic characterization of Italian chicken breeds by next-generation sequencing discovery and array genotyping. Poult. Sci..

[20] Ozdemir D., Maretto F., Cassandro M. (2016). Comparison of genetic diversity of Turkish and Italian local chicken breeds for further conservation strategies. Eur. Poult. Sci..

[21] Ozdemir D., Ozdemir E.D., De Marchi M., Cassandro M. (2013). Conservation of local Turkish and Italian chicken breeds: A case study. Ital. J. Anim. Sci..

[22] Zanetti E., De Marchi M., Abbadi M., Cassandro M. (2011). Variation of genetic diversity over time in local Italian chicken breeds undergoing in situ conservation. Poult. Sci..

[23] Zanetti E., Molette C., Chambon C., Pinguet J., Remignon H., Cassandro M. (2011). Using 2-DE for the differentiation of local chicken breeds. Proteomics.

[24] Sironi L., Lazzari B., Ramelli P., Stella A., Mariani P. (2008). Avian TAP genes: Detection of nucleotide polymorphisms and comparative analysis across species. Genet. Mol. Res..

[25] Strillacci M.G., Marelli S.P., Cozzi M.C., Colombo E., Polli M., Gualtieri M., Cristalli A., Pignattelli P., Longeri M., Cavalchini L.G. (2009). Italian autochthonous chicken breeds conservation: Evaluation of biodiversity in Valdarnese Bianca breed (*Gallus gallus domesticus*). Avian Biol. Res..

[26] Cozzi M.C., Colombo E., Zaniboni L., Madeddu M., Mosca F., Strillacci M.G., Longeri M., Bagnato A., Cerolini S. (2017). Phenotypic and genetic characterization of the Italian bantam chicken breed Mericanel della Brianza. Livest. Sci..

[27] Gliozzi T.M., Zaniboni L., Cerolini S. (2011). DNA fragmentation in chicken spermatozoa during cryopreservation. Theriogenology.

[28] Strillacci M.G., Gorla E., Rios-Utrera A., Vega-Murillo V.E., Montano-Bermudez M., Garcia-Ruiz A., Cerolini S., Roman-Ponce S.I., Bagnato A. (2019). Copy number variation mapping and genomic variation of autochthonous and commercial turkey populations. Front. Genet..

[29] Colombo E., Strillacci M.G., Cozzi M.C., Madeddu M., Mangiagalli M.G., Mosca F., Zaniboni L., Bagnato A., Cerolini S. (2014). Feasibility study on the FAO chicken microsatellite panel to assess genetic variability in the turkey (*Meleagris gallopavo*). Ital. J. Anim. Sci..

[30] Castellini C., Mugnai C., Moscati L., Mattioli S., Amato M.G., Mancinelli A.C., Dal Bosco A. (2016). Adaptation to organic rearing system of eight different chicken genotypes: Behavior, welfare and performance. Ital. J. Anim. Sci..

[31] Mugnai C., Sossidou E.N., Dal Bosco A., Ruggeri S., Mattioli S., Castellini C. (2014). The effects of husbandry system on the grass intake and egg nutritive characteristics of laying hens. J. Sci. Food Agric..

[32] Dal Bosco A., Mugnai C., Ruggeri S., Mattioli S., Castellini C. (2012). Fatty acid composition of meat and estimated indices of lipid metabolism in different poultry genotypes reared under organic system. Poult. Sci..

[33] Mugnai C., Dal Bosco A., Castellini C. (2009). Effect of rearing system and season on the performance and egg characteristics of Ancona laying hens. Ital. J. Anim. Sci..

[34] Castillo A., Marzoni M., Chiarini R., Romboli I. Razza Ancona: Indagini preliminari sulle caratteristiche riproduttive. Proceedings of the Convegno Nazionale “Parliamo di…… Globalizzazione e Diversificazione in Zootecnica”.

[35] Soglia D., Sartore S., Maione S., Schiavone A., Dabbou S., Nery J., Zaniboni L., Marelli S., Sacchi P., Rasero R. (2020). Growth performance analysis of two Italian slow-growing chicken breeds: Bianca di Saluzzo and Bionda Piemontese. Animals.

[36] Ferrante V., Mugnai C., Ferrari L., Marelli S.P., Spagnoli E., Lolli S. (2016). Stress and reactivity in three Italian chicken breeds. Ital. J. Anim. Sci..

[37] Marelli S.P., Terova G., Cozzi M.C., Lasagna E., Sarti F.M., Cavalchini L.G. (2010). Gene expression of hepatic glucocorticoid receptor NR3C1 and correlation with plasmatic corticosterone in Italian chickens. Anim. Biotechnol..

[38] Rizzi C. (2020). Yield performance, laying behavior traits and egg quality of purebred and hybrid hens reared under outdoor conditions. Animals.

[39] Rizzi C., Verdiglione R. (2015). Testicular growth and comb and wattles development in three Italian chicken genotypes reared under free-range conditions. Ital. J. Anim. Sci..

[40] Rizzi C., Chiericato G.M. (2010). Chemical composition of meat and egg yolk of hybrid and Italian breed hens reared using an organic production system. Poult. Sci..

[41] Rizzi C., Baruchello M., Chiericato G.M. (2009). Effect of sex on slaughter performance and meat quality of Ermellinata di Rovigo chickens. Ital. J. Anim. Sci..

[42] Rizzi C., Marangon A., Chiericato G.M. (2007). Effect of genotype on slaughtering performance and meat physical and sensory characteristics of organic laying hens. Poult. Sci..

[43] Marzoni M., Castillo A., Franzoni A., Nery J., Fortina R., Romboli I., Schiavone A. (2020). Effects of Dietary Quebracho Tannin on Performance Traits and Parasite Load in an Italian Slow-Growing Chicken (White Livorno Breed). Animals.

[44] Di Rosa A.R., Chiofalo B., Lo Presti V., Chiofalo V., Liotta L. (2020). Egg quality from Siciliana and Livorno Italian autochthonous chicken breeds reared in organic system. Animals.

[45] Marzoni M., Castillo A., Chiarini R., Romboli I. Indagine preliminare sulle prestazioni produttive di una razza avicola autoctona: La razza Livorno. Proceedings of the Parliamo di …allevamenti alternativi e valorizzazione del territorio.

[46] Zaniboni L., Cassinelli C., Mangiagalli M.G., Gliozzi T.M., Cerolini S. (2014). Pellet cryopreservation for chicken semen: Effects of sperm working concentration, cryoprotectant concentration, and equilibration time during in vitro processing. Theriogenology.

[47] Madeddu M., Zaniboni L., Mangiagalli M.G., Cassinelli C., Cerolini S. (2013). Egg related parameters affecting fertility and hatchability in the Italian bantam breed Mericanel della Brianza. Anim. Rep. Sci..

[48] Cerolini S., Madeddu M., Zaniboni L., Cassinelli C., Mangiagalli M.G., Marelli S.P. (2010). Breeding performance in the Italian chicken breed Mericanel della Brianza. Ital. J. Anim. Sci..

[49] Cerolini S., Vasconi M., Sayed A.A., Iaffaldano N., Mangiagalli M.G., Pastorelli G., Moretti V.M., Zaniboni L., Mosca F. (2019). Free-range rearing density for male and female Milanino chickens: Carcass yield and qualitative meat traits. J. Appl. Poult. Res..

[50] Mosca F., Zaniboni L., Iaffaldano N., Sayed A.A., Mangiagalli M.G., Pastorelli G., Cerolini S. (2019). Free-range rearing density for male and female Milanino chickens: Growth performance and stress markers. J. Appl. Poult. Res..

[51] Mosca F., Kuster C.A., Stella S., Farina G., Madeddu M., Zaniboni L., Cerolini S. (2016). Growth performance, carcass characteristics and meat composition of Milanino chickens fed on diets with different protein concentrations. Br. Poult. Sci..

[52] Mosca F., Madeddu M., Mangiagalli M.G., Colombo E., Cozzi M.C., Zaniboni L., Cerolini S. (2015). Bird density, stress markers and growth performance in the Italian chicken breed Milanino. J. Appl. Poult. Res..

[53] Zanon A., Beretti V., Superchi P., Zambini E.M., Sabbioni A. Physico-chemical characteristics of eggs from two Italian autochthonous chicken breeds: Modenese and Romagnolo. Proceedings of the WPSA XII European Poultry Conference.

[54] Minieri S., Buccioni A., Serra A., Galigani I., Pezzati A., Rapaccini S., Antongiovanni M. (2016). Nutritional characteristics and quality of eggs from laying hens fed on a diet supplemented with chestnut tannin extract (*Castanea sativa* Miller). Br. Poult. Sci..

[55] Zotte A.D., Tasoniero G., Baldan G., Cullere M. (2019). Meat quality of male and female Italian Padovana and Polverara slow-growing chicken breeds. Ital. J. Anim. Sci..

[56] Rizzi C. (2019). Growth and slaughtering performance, carcass fleshiness and meat quality according to the plumage colour in Padovana male chickens slaughtered at 18 weeks of age. Ital. J. Anim. Sci..

[57] Tasoniero G., Cullere M., Baldan G., Zotte A.D. (2018). Productive performances and carcase quality of male and female Italian Padovana and Polverara slow-growing chicken breeds. Ital. J. Anim. Sci..

[58] Rizzi C., Contiero B., Cassandro M. (2013). Growth patterns of Italian local chicken populations. Poult. Sci..

[59] Zanetti E., De Marchi M., Dalvit C., Molette C., Remignon H., Cassandro M. (2010). Carcass characteristics and qualitative meat traits of three Italian local chicken breeds. Br. Poult. Sci..

[60] Zotte A.D., Ricci R., Cullere M., Serva L., Tenti S., Marchesini G. (2020). Research Note: Effect of chicken genotype and white striping-wooden breast condition on breast meat proximate composition and amino acid profile. Poult. Sci..

[61] Rizzi C., Baruchello M., Chiericato G.M. (2009). Slaughter performance and meat quality of three Italian chicken breeds. Ital. J. Anim. Sci..

[62] Sirri F., Zampiga M., Soglia F., Meluzzi A., Cavani C., Petracci M. (2018). Quality characterization of eggs from Romagnola hens, an Italian local breed. Poult. Sci..

[63] Italian Veterinary Service (2019). Italian Registry of Animals.

[64] Bittante G. (2011). Italian animal genetic resources in the Domestic Animal Diversity Information System of FAO. Ital. J. Anim. Sci..

[65] Cendron F., Perini F., Mastrangelo S., Tolone M., Criscione A., Bordonaro S., Iaffaldano N., Castellini C., Marzoni M., Buccioni A. (2020). Genome-wide SNP analysis reveals the population structure and the conservation status of 23 Italian chicken breeds. Animals.

[66] TuBavI Project Conservation of Biodiversity in Italian Poultry Breeds. https://www.pollitaliani.it/en/.

[67] De Marco M., Dalmasso A., Bottero M.T., Pattono D., Sponza S., Sacchi P., Rasero R., Sartore S., Soglia D., Giacobini M. Local poultry breed assessment in Piemonte (north-west Italy). Proceedings of the 8th European Symposium on Poultry Genetics.

[68] Microsoft Corporation (2019). Microsoft Excel, 2019.

[69] SAS Institute Inc (2002). JMP Statistical Discovery, 5.0.1..

[70] Swan S.E.J., Sonaiya E. (2007). Small Scale Poultry Production: Technical Guide.

[71] Lazzaroni C., Moriano G. The role of Producers’ Association in the valorisation of traditional products: An Italian North-West poultry and rabbit breeds Consortium. Proceedings of the Mediterranean Livestock Production: Uncertainties and Opportunities, 2nd Seminar of Mediterranean Livestock Farming Network.

[72] Elkhoraibi C., Blatchford R.A., Pitesky M.E., Mench J.A. (2014). Backyard chickens in the United States: A survey of flock owners. Poult. Sci..

[73] Abdelqader A., Wollny C.B.A., Gauly M. (2007). Characterization of local chicken production systems and their potential under different levels of management practice in Jordan. Trop. Anim. Health Prod..

[74] Slow Food Foundation for Biodiversity Morozzo Capon. https://www.fondazioneslowfood.com/it/presidi-slow-food/cappone-di-morozzo/.

[75] Scott A.B., Singh M., Toribio J.A., Hernandez-Jover M., Barnes B., Glass K., Moloney B., Lee A., Groves P. (2018). Comparisons of management practices and farm design on Australian commercial layer and meat chicken farms: Cage, barn and free range (vol 12, e0188505, 2017). PLoS ONE.

[76] Gondwe T.N., Wollny C.B.A. (2007). Local chicken production system in Malawi: Household flock structure, dynamics, management and health. Trop. Anim. Health Prod..

[77] Pérez B.A., Polanco E.G. (2003). La avicultura de traspatio en zonas campesinas de la provincia de Villa Clara, Cuba. Livest. Res. Rural Dev..

[78] Rodríguez J.C., Allaway C.E., Wassink G.J., Segura J.C., Rivera T. (1996). Estudio de la avicultura de traspatio en el municipio de Dzununcán, Yucatán. Vet. Méx..

[79] Popy F.Y., Chowdhury Q.M.M., Alam S., Roy S., Dipta P.M., Ahmed J. (2018). Backyard Poultry Management and Production System at Barlekha Upazila, Moulvibazar. Int. J. Sci. Bus..

[80] Cartoni-Mancinelli A., Franzoni A., Dal Bosco A., Schiavone A., Mannelli F., Marzoni M., Castellini C. (2020). Distribution and consistency of Ancona and Livorno poultry breed in Central Italy. Ital. J. Anim. Sci..

[81] Lockhart C.Y., Stevenson M.A., Rawdon T.G. (2010). A cross-sectional study of ownership of backyard poultry in two areas of Palmerston North, New Zealand. Vet. J..

[82] Karabozhilova I., Wieland B., Alonso S., Salonen L., Hasler B. (2012). Backyard chicken keeping in the Greater London Urban Area: Welfare status, biosecurity and disease control issues. Br. Poult. Sci..

[83] Van Staaveren N., Decina C., Baes C.F., Widowski T.M., Berke O., Harlander-Matauschek A. (2018). A description of laying hen husbandry and management practices in Canada. Animals.

[84] Beam A., Garber L., Sakugawa J., Kopral C. (2013). Salmonella awareness and related management practices in US urban backyard chicken flocks. Prev. Vet. Med..

[85] Burns T.E., Ribble C., McLaws M., Kelton D., Stephen C. (2013). Perspectives of an underrepresented stakeholder group, backyard flock owners, on poultry health and avian influenza control. J. Risk Res..

[86] Garber L., Hill G., Rodriguez J., Gregory G., Voelker L. (2007). Non-commercial poultry industries: Surveys of backyard and gamefowl breeder flocks in the United States. Prev. Vet. Med..

