# Supplementary material for: Overview of Native Chicken Breeds in Italy: Conservation Status and Rearing Systems in Use
Source: Animals (Basel). 2021 Feb 12;11(2):490. doi: 10.3390/ani11020490 (PMC7917728; doi:10.3390/ani11020490)
Supplement: Supplementary file 1 [file animals-11-00490-s001.pdf]

animals

MDPI

Article

Tables S

Supplementary Materials: Table S1: Manpower involved in the care and management of flocks: responses from all breeders and divided according to breeder category, Table S2: Environmental housing conditions adopted: responses from all breeders and divided according to breeder category, Table S3: Pen cover and ground vegetation: responses from all breeders and divided according to breeder category, Table S4: Litter management: responses from all breeders and divided according to breeder category, Table S5: Pen furnishings: responses from all breeders and divided according to breeder category, Table S6: Self-production of feed primary materials: responses from all breeders and divided according to breeder category, Table S7: Flock vaccinations and medical treatments performed: responses from all breeders and divided according to breeder category, Table S8: Farm location and presence of a cold storage room for dead animals: responses from all breeders and divided according to breeder category, Table S9: Frequency of interventions against rodents: responses from all breeders and divided according to breeder category.

Table S1. Manpower involved in the care and management of flocks: responses from all breeders and divided according to breeder category.

	All Breeders		Farmers		Fancy Breeders		
**Variable**	**n**	**%**	**n**	**%**	**n**	**%**	χ^{2} [1]
Employees	(n = 105)		(n = 72)		(n = 33)		
Family members	80 ^{A}	77	48 ^{A}	67	32 ^{A}	97	**
External personnel	11 ^{B}	10	10 ^{B}	14	1 ^{B}	3	NS
Family members + external personnel	14 ^{B}	13	14 ^{B}	19	0 ^{B}	0	**
Family members (n)	(n = 78)		(n = 57)		(n = 21)		
< than 5	74 ^{A}	95	53 ^{A}	93	21 ^{A}	100	NS
5 or more	4 ^{B}	5	4 ^{B}	7	0 ^{B}	0	NS
External personnel (n)	(n = 25)		(n = 24)		(n = 1)		
< than 5	15 ^{A}	60	15 ^{A}	63	0	0	NS
Between 5 - 10	8 ^{A,B}	32	7 ^{B}	29	1	100	NS
> than 10	2 ^{B}	8	2 ^{B}	8	0	0	NS

[1] Chi square test for a single variable between the two breeder categories, i.e. within row comparisons; significance levels: ** $p < 0.01$; NS, non-significant ($p > 0.05$). $^{A-B}$ Observations with different superscripts within the column are significantly different (χ^2-test $p < 0.01$).

Table S2. Environmental housing conditions adopted: responses from all breeders and divided according to breeder category.

Variable	All Breeders		Farmers		Fancy Breeders		χ^2 [1]
	n	%	n	%	n	%	
Heating system	(n = 77)		(n = 62)		(n = 15)		
Yes	38	49	36	58	2 [B]	13	**
No	39	51	26	42	13[A]	87	**
Cooling system	(n = 77)		(n = 62)		(n = 15)		
Yes	2 [B]	3	2 [B]	3	0 [B]	0	NS
No	75 [A]	97	60 [A]	97	15 [A]	100	NS
Ventilation system	(n = 77)		(n = 62)		(n = 15)		
Environmental conditions	73 [A]	95	58 [A]	94	15 [A]	100	NS
Forced air system	0 [B]	0	0 [B]	0	0 [B]	0	NS
Combined system	4 [B]	5	4 [B]	6	0 [B]	0	NS
Lighting system	(n = 77)		(n = 62)		(n = 14)		
Environmental conditions	60 [A]	78	48 [A]	78	12 [A]	86	NS
Artificial lighting	2 [C]	3	2 [C]	3	0 [B]	0	NS
Combined system	14 [B]	18	12 [B]	19	2 [B]	14	NS
Temperature measurement	(n = 77)		(n = 62)		(n = 15)		
Yes	6 [B]	8	5 [B]	8	1 [B]	7	NS
No	71[A]	92	57 [A]	92	14 [A]	93	NS
RH measurement	(n = 77)		(n = 62)		(n = 15)		
Yes	4 [B]	5	3 [B]	5	1 [B]	7	NS
No	73 [A]	95	59 [A]	95	14 [A]	93	NS
Air quality measurement	(n = 77)		(n = 62)		(n = 15)		
Yes	1 [B]	1	1 [B]	2	0 [B]	0	NS
No	76 [A]	99	61 [A]	98	15 [A]	100	NS

[1] Chi square test for a single variable between the two breeder categories, i.e. within row comparisons; significance levels: ** $p < 0.01$; NS, non-significant ($p > 0.05$). [A-C] Observations with different superscripts within the column are significantly different (χ^2-test $p < 0.01$).

Table S3. Pen cover and ground vegetation: responses from all breeders and divided according to breeder category.

	All Breeders		Farmers		Fancy Breeders		
**Variable**	**n**	**%**	**n**	**%**	**n**	**%**	$\chi^{2\,1}$
Cover	(n = 105)		(n = 68)		(n = 37)		
Yes	45	43	21 ^{B}	31	24 ^{a}	65	**
No	60	57	47 ^{A}	69	13 ^{b}	35	**
Cover material	(n = 23)		(n = 15)		(n = 8)		
Netting	9 ^{A}	39	9 ^{A}	60	0 ^{B}	0	**
Fabric canopy	12 ^{A}	52	6 ^{A}	40	6 ^{A}	75	NS
Netting & fabric canopy	2 ^{B}	9	0 ^{B}	0	2 ^{A,B}	25	NS
Vegetation	(n = 87)		(n = 59)		(n = 28)		
Meadow	14 ^{B}	16	11 ^{B}	19	3 ^{B,C}	11	NS
Bushes	3 ^{C}	3	3 ^{C}	5	0^{C}	0	NS
Trees	30 ^{A}	35	22 ^{A}	37	8 ^{A,B}	28	NS
Combination of the above	40 ^{A}	46	23 ^{A}	39	17 ^{A}	61	NS
Type of meadow	(n = 51)		(n = 34)		(n = 17)		
Perennial	18 ^{A}	35	12 ^{A}	35	6 ^{A}	35	NS
Polyphyletic	27 ^{A}	53	18 ^{A}	53	9 ^{A}	53	NS
Graminaceous	3 ^{B}	6	1 ^{B}	3	2 ^{A,B}	12	NS
Alfalfa	3 ^{B}	6	3 ^{B}	9	0 ^{B}	0	NS

[1] Chi square test for a single variable between the two breeder categories, i.e. within row comparisons; significance levels: ** $p < 0.01$; NS, non-significant ($p > 0.05$). $^{A-C}$ Observations with different superscripts within the column are significantly different (χ2-test $p < 0.01$). $^{a-b}$ Observations with different superscripts within the column are significantly different (χ^2-test, $p < 0.05$).

Table S4. Litter management: responses from all breeders and divided according to breeder category.

	All Breeders		Farmers		Fancy Breeders		
**Variable**	**n**	**%**	**n**	**%**	**n**	**%**	$\chi^{2\,1}$
Addition of additives	(n = 71)		(n = 56)		(n = 15)		
Yes	2 ^{B}	3	1 ^{B}	2	1 ^{B}	7	NS
No	69 ^{A}	97	55 ^{A}	98	14 ^{A}	93	NS
Flip over of the litter	(n = 70)		(n = 56)		(n = 14)		
Yes	11 ^{B}	16	4 ^{B}	7	7	50	**
No	59 ^{A}	84	52 ^{A}	93	7	50	**

[1] Chi square test for a single variable between the two breeder categories, i.e. within row comparisons; significance levels: ** $p < 0.01$; NS, non-significant ($p > 0.05$). $^{A-C}$ Observations with different superscripts within the column are significantly different (χ2-test $p < 0.01$).

Table S5. Pen furnishings: responses from all breeders and divided according to breeder category.

	All Breeders		Farmers		Fancy Breeders		
**Variable**	**n**	**%**	**n**	**%**	**n**	**%**	χ^{2} [1]
Water distribution	(n = 120)		(n = 75)		(n = 45)		
Manual	84 ^{A}	70	53 ^{A}	70	31 ^{A}	69	NS
Automatic	29 ^{B}	24	17 ^{B}	23	12 ^{B}	27	NS
Both	7 ^{C}	6	5 ^{C}	7	2 ^{C}	4	NS
Feed distribution	(n = 119)		(n = 75)		(n = 44)		
Manual	109 ^{A}	92	69 ^{A}	92	40 ^{A}	91	NS
Automatic	10 ^{B}	8	6 ^{B}	8	4 ^{B}	9	NS
Both	0 ^{C}	0	0 ^{C}	0	0^{C}	0	
Silos	(n = 97)		(n = 66)		(n = 31)		
Yes	29 ^{B}	30	23 ^{B}	35	6 ^{B}	19	NS
No	68 ^{A}	70	43 ^{A}	65	25 ^{A}	81	NS
Nests	(n = 119)		(n = 75)		(n = 44)		
Yes	112 ^{A}	94	69 ^{A}	92	43 ^{A}	98	NS
No	7 ^{B}	6	6 ^{B}	8	1 ^{B}	2	NS
Nests	(n = 104)		(n = 64)		(n = 40)		
Single	27 ^{B}	26	15 ^{B}	24	12 ^{B}	30	NS
Group	72 ^{A}	69	47 ^{A}	73	25 ^{A}	63	NS
Both	5 ^{C}	5	2 ^{C}	3	3 ^{C}	7	NS

[1] Chi square test for a single variable between the two breeder categories, i.e. within row comparisons; significance levels: NS, non-significant ($p > 0.05$). $^{A\text{-}C}$ Observations with different superscripts within the column are significantly different (χ^2-test $p < 0.01$).

Table S6. Self-production of feed primary materials: responses from all breeders and divided according to breeder category.

Variable	All Breeders		Farmers		Fancy Breeders		χ^{2} [1]
	**n**	**%**	**n**	**%**	**n**	**%**	
Self-production of Primary Materials[2]	(n = 74)		(n = 55)		(n = 19)		
Maize	65 [A]	88	49 [A]	89	16 [a]	84	NS
Wheat	38 [B]	51	27 [B]	49	11 [a,b]	58	NS
Barley	33 [B,C]	45	22 [B,C]	40	11 [a,b]	58	NS
Bran	22 [C]	30	15 [C]	27	7 [b]	37	NS
Soybean	24 [C]	32	14 [C]	25	10 [a,b]	53	*
Other	26 [B,C]	35	18 ,[BC]	33	8 [b]	42	NS

[1] Chi square test for a single variable between the two breeder categories, i.e. within row comparisons; significance levels: * $p < 0.05$; NS, non-significant ($p > 0.05$). [A-C] Observations with different superscripts within the column are significantly different (χ^2-test $p < 0.01$). [a-b] Observations with different superscripts within the column are significantly different (χ^2-test, $p < 0.05$).

Table S7. Flock vaccinations and medical treatments performed: responses from all breeders and divided according to breeder category.

	All Breeders		Farmers		Fancy Breeders		
**Variable**	**n**	**%**	**n**	**%**	**n**	**%**	χ^{2} [1]
Marek's Disease vaccination	(n = 53)		(n = 34)		(n = 19)		
Yes	36 ^{A}	68	25 ^{A}	74	11	58	NS
No	17 ^{B}	32	9 ^{B}	26	8	42	NS
Newcastle Disease vaccination	(n=72)		(n=41)		(n=31)		
Yes	72 ^{A}	100	41 ^{A}	100	31 ^{A}	100	NS
No	0 ^{B}	0	0 ^{B}	0	0 ^{B}	0	NS
Fowl Pox vaccination	(n = 47)		(n = 30)		(n = 17)		
Yes	26	55	21 ^{A}	70	5 ^{b}	29	**
No	21	45	9 ^{B}	30	12 ^{a}	71	**
Infectious Bronchitis vaccination	(n = 45)		(n = 28)		(n = 17)		
Yes	22	49	14	50	8	47	NS
No	23	51	14	50	9	53	NS
Infect. Bursal Disease vaccination	(n = 40)		(n = 28)		(n = 12)		
Yes	15 ^{B}	38	14	50	1 ^{B}	8	*
No	25 ^{A}	62	14	50	11 ^{A}	92	*
Infectious Coriza vaccination	(n=30)		(n=17)		(n=13)		
Yes	5 ^{B}	17	3 ^{B}	18	2 ^{B}	15	NS
No	25 ^{A}	83	14 ^{A}	82	11 ^{A}	85	NS
Coccidiosis vaccination	(n = 43)		(n = 29)		(n = 14)		
Yes	16 ^{b}	37	14	48	2 ^{B}	14	**
No	27 ^{a}	63	15	52	12 ^{A}	86	NS
Coccidiosis treatment	(n = 82)		(n = 52)		(n = 30)		
Yes	44	54	27	52	17	57	NS
No	38	46	25	48	13	43	NS
Worms treatment	(n = 96)		(n = 61)		(n = 35)		
Yes	37 ^{B}	39	21 ^{B}	34	16	46	NS
No	59 ^{A}	61	40 ^{A}	66	19	54	NS
Ectoparasites treatment	(n = 58)		(n = 36)		(n = 22)		
Yes	38 ^{A}	66	26 ^{A}	72	12	55	NS
No	20 ^{B}	34	10 ^{B}	28	10	45	NS

[1] Chi square test for a single variable between the two breeder categories, i.e. within row comparisons; significance levels: ** $p < 0.01$; * $p < 0.05$; NS, non-significant ($p > 0.05$). A-B Observations with different superscripts within the column are significantly different (χ2-test $p < 0.01$). a-b Observations with different superscripts within the column are significantly different (χ^2-test, $p < 0.05$).

Table S8. Farm location and presence of a cold storage room for dead animals: responses from all breeders and divided according to breeder.

	All Breeders		Farmers		Fancy Breeders		
**Variable**	**n**	**%**	**n**	**%**	**n**	**%**	χ^2 [1]
Near major roads	(n = 119)		(n = 75)		(n = 44)		
Yes	21 [B]	18	12 [B]	16	9 [B]	20	NS
No	98 [A]	82	63 [A]	84	35 [A]	78	NS
Near industrial areas	(n = 118)		(n=75)		(n=43)		
Yes	10 [B]	8	5 [B]	67	5 [B]	12	NS
No	108 [A]	92	70 [A]	93	38 [A]	88	NS
Cold storage room for dead birds	(n = 110)		(n = 74)		(n = 36)		
Yes	36 [B]	33	32	43	4 [B]	11	**
No	74 [A]	67	42	57	32 [A]	89	NS

[1] Chi square test for a single variable between the two breeder categories, i.e. within row comparisons; significance levels: ** $p < 0.01$; NS, non-significant ($p > 0.05$). A-B Observations with different superscripts within the column are significantly different (χ^2-test $p < 0.01$).

Table S9. Frequency of interventions against rodents: responses from all breeders and divided according to breeder category.

	All Breeders		Farmers		Fancy Breeders		
**Variable**	**n**	**%**	**n**	**%**	**n**	**%**	χ^2 [1]
Intervention frequency in feed storeroom	(n = 33)		(n = 26)		(n = 7)		
At least every 15 days	6 [a,b]	18	4 [b]	15	2	29	NS
Every 15–30 days	4 [b]	12	3 [b]	12	1	14	NS
Every 30–60 days	14 [a]	43	12 [a]	46	2	29	NS
< than every 60 days	9 [a,b]	27	7 [a,b]	27	2	28	NS
Intervention frequency in the chicken shed	(n = 29)		(n = 21)		(n = 8)		
At least every 15 days	5	17	3 [b]	14	2	25	NS
Every 15–30 days	4	14	3 [b]	14	1	12	NS
Every 30–60 days	12	41	10 [a]	48	2	25	NS
< than every 60 days	8	28	5 [a,b]	24	3	38	NS

[1] Chi square test for a single variable between the two breeder categories, i.e. within row comparisons; significance levels: NS, non-significant ($p > 0.05$). a-b Observations with different superscripts within the column are significantly different (χ^2-test, $p < 0.05$).
